# Machine Learning-Based Prediction of Mononuclear Cell Collection Efficiency for CAR-T Manufacturing

**DOI:** 10.1007/s11596-026-00237-1

**Published:** 2026-08-05

**Authors:** Wei Xie, Lin Liu, Jin-hui Shu, Meng-yi Du, Heng Mei

**Affiliations:** 1 Institute of Hematology, Union Hospital, Tongji Medical College, Huazhong University of Science and Technology, Wuhan, 430022 China; 2 Hubei Clinical Medical Center of Cell Therapy for Neoplastic Disease, Wuhan, 430022 China

**Keywords:** CAR-T therapy, Leukapheresis, Collection efficiency, Mononuclear cells, Machine learning, XGBoost

## Abstract

**Objective:**

To develop and internally validate an interpretable machine-learning model using routine precollection variables to predict mononuclear cell (MNC) collection efficiency (CE) during leukapheresis for CAR-T manufacturing.

**Methods:**

This retrospective study included 206 consecutive patients who underwent leukapheresis between 2022 and 2025. Candidate variables were screened with Pearson correlation, Boruta, recursive feature elimination, and variance-threshold filters. Six supervised algorithms were trained with nested cross-validation, using an inner fivefold loop for tuning and an outer tenfold loop repeated three times for performance estimation. Bootstrap resampling (1000 iterations) generated confidence intervals, and SHAP analysis was used for interpretation.

**Results:**

Extreme gradient boosting (XGBoost) showed the best continuous prediction performance, with an *R*^2^ of 0.67 (95% CI: 0.64–0.70), Pearson *r* = 0.82, RMSE = 12.4%, and MAE = 9.8%. In the secondary triage analysis, observed high CE was defined as CE ≥ 60% (33/206, 16.0%). The predicted CE score discriminated high CE with an AUC of 0.84. A predicted-CE threshold of ≥ 50% yielded sensitivity of 84.8%, specificity of 97.1%, PPV of 84.8%, and NPV of 97.1%. The leading predictors were precollection lymphocyte count, lymphocyte/WBC ratio, age, body surface area, and monocyte count.

**Conclusion:**

Routine precollection variables estimated MNC CE with good internal validity and may support individualized leukapheresis planning before CAR-T manufacturing.

**Supplementary Information:**

The online version contains supplementary material available at 10.1007/s11596-026-00237-1.

## Introduction

Chimeric antigen receptor T-cell (CAR-T) therapy has transformed the treatment landscape for relapsed or refractory hematologic malignancies [1,2]. Manufacturing, however, depends on the quality and quantity of autologous T cells collected before production [3]. Leukapheresis quality remains a practical limitation because patient status, blood count, and collection conditions vary substantially at the time of harvest [4]. Collection efficiency (CE), defined as the proportion of target mononuclear cells (MNCs) harvested relative to the processed cellular load, is therefore an operational metric for leukapheresis planning [5]. Suboptimal collections can require procedure adjustment, repeated collection, or manufacturing contingency planning [6].

Clinical decisions regarding leukapheresis scheduling and parameter settings still rely heavily on local practice, collection-center experience, and basic hematologic thresholds [7]. Italian expert panel recommendations similarly emphasize standardized assessment before CAR-T leukapheresis but do not provide an individualized CE prediction model [8]. Existing T-cell collection prediction studies have used conventional algorithms or mathematical models rather than flexible nonlinear estimators [9,10]. Age-related immune variation and immune reconstitution may further modify collection behavior in ways that simple linear models may not capture [11].

Recent machine-learning studies in hematology illustrate how nonlinear models can summarize multivariable clinical patterns [12]. Similar approaches have been applied to flow cytometry diagnosis and hematopoietic transplantation outcomes [13,14]. For collection planning, however, the output must remain interpretable enough to support a clinical decision rather than function as an opaque score [15]. We therefore aligned the revised reporting with current prediction-model reporting guidance for regression and machine-learning methods [16].

Here, we developed and internally validated an interpretable model for MNC CE using variables available before collection. We compared extreme gradient boosting (XGBoost) with linear, penalized, tree-based, and simple clinical baseline models; used ensemble feature selection to reduce the candidate predictor set; and translated the continuous prediction into risk strata that could be checked against observed CE distributions in the same cohort.

## Materials and Methods

### Study Design and Population

This retrospective single-center study included consecutive patients who underwent leukapheresis for CAR-T manufacturing between 2022 and 2025. Eligibility criteria were age ≥ 15 years, a diagnosis of hematologic malignancy, and the availability of precollection laboratory data. Patients were excluded if they were enrolled in another interventional trial, had uncontrolled active infection, or had hemodynamic instability precluding standard apheresis. The study was approved by the Medical Ethics Committee of Union Hospital, Tongji Medical College, Huazhong University of Science and Technology (No. 2024-IEC-0354) and was conducted in accordance with the Declaration of Helsinki.

### Outcome Definition

The primary outcome was the clinically recorded MNC CE. To make the calculation transparent, CE was described using the CE2-style denominator correction recommended in CAR-T leukapheresis guidance and recent collection-efficiency reports [8, 17,18]:

$${\mathrm{CE}}\left( \% \right) = \left[ {\left( {{\mathrm{Cproduct}} \times {\mathrm{Vproduct}}} \right)/\left( {{\mathrm{Cpre}} \times \left( {\text{Vprocessed - VACD}} \right)} \right)} \right] \times 100$$

In this equation, Cproduct denotes the product MNC concentration, Vproduct denotes the product volume, Cpre denotes the pre-apheresis MNC concentration, Vprocessed denotes the processed blood volume, and VACD denotes the acid-citrate-dextrose (ACD) volume. Subtracting the VACD avoids inflating the denominator with the anticoagulant volume.

Patient-level ACD volumes were not consistently recorded in the analytic dataset. Therefore, the primary analysis retained the recorded clinical CE values, with values exceeding 100% before capping (n = 8) retained for quality control and capped at 100% to maintain physiological plausibility. To test whether correcting the ACD denominator would substantially change the findings, we performed an ACD-adjusted sensitivity analysis using the standard 12:1 whole blood:ACD assumption rather than replacing the primary endpoint with partially reconstructed values.

### Data Preprocessing and Missing Data

To ensure data quality, variable names were standardized, and duplicate records were removed. Missingness was inspected by variable and by case before imputation; the highest variable-level missingness rate was 3.4%, and no key analytic variable exceeded 5% missingness (Table S1). Missing values were imputed using multiple imputation by chained equations (MICE) [19], with five imputed datasets and 20 chained iterations. Predictive mean matching was used for continuous variables, and multinomial logistic imputation was used for categorical variables. The imputed dataset was used for primary model development, and listwise deletion, mean imputation, and random-forest imputation were evaluated as internal robustness checks rather than substitutes for external validation.

### Feature Engineering and Selection

We derived clinically relevant features to capture physiological dynamics, including body surface area (BSA) and hematologic ratios. BSA was calculated using the Du Bois formula [20].

The cellular ratios included the lymphocyte/white blood cell (WBC) ratio, platelet/WBC ratio, and hemoglobin/hematocrit ratio.

To reduce the candidate predictor set, we used an ensemble feature-selection strategy that integrated four complementary approaches: Pearson correlation screening (two-sided *P* < 0.05), Boruta random-forest feature selection [21] (500 trees and maxRuns = 500), recursive feature elimination [22] using repeated cross-validation and RMSE as the scoring criterion, and near-zero-variance filtering. Predictors retained by at least three of the four methods were entered into final model training. Imputation strategies, feature-selection thresholds, and alternative preprocessing procedures were evaluated as internal robustness procedures (Table S3); because this was a single-center retrospective analysis, the resulting performance estimates may remain optimistic. The exact retained and excluded variables are listed in Table S4.

### Model Development

We evaluated six supervised learning algorithms: linear regression, ridge regression, LASSO regression, elastic net, random forest, and XGBoost [23]. The complete hyperparameter search spaces are provided in Table S2. The penalized linear models were tuned over lambda values from 1 × 10⁻^4^ to 1 × 10; the elastic net additionally used alpha values from 0.1 to 0.9. The random forest algorithm used 500 trees with mtry values of 2, 4, 6, 8, and 10 and minimum node sizes of 1, 3, and 5. XGBoost was tuned over 100, 200, 300, and 500 boosting rounds; maximum depths of 3, 6, and 9; and learning rates of 0.01, 0.1, and 0.3, respectively.

### Model Evaluation and Validation

Because the sample size was modest (n = 206), model development and performance estimation were conducted within a nested cross-validation framework rather than a single train/test split [24].

The inner loop used fivefold cross-validation for hyperparameter tuning.

The outer loop used tenfold cross-validation to provide an internal estimate of generalization performance; the outer tenfold procedure was repeated three times only for the stability summaries shown in Supplementary Materials. Bootstrap resampling (1000 iterations) was used to derive confidence intervals for R^2^, RMSE, MAE, and discrimination of observed CE ≥ 60%. Because model development and validation were performed within one center, these estimates may still be optimistic relative to prospective external validation [25].

### Statistical Analysis

Baseline characteristics were compared using Welch’s ANOVA for continuous variables and chi-square tests for categorical variables. High-efficiency triage was evaluated by using the continuous predicted CE score to discriminate observed CE ≥ 60%; sensitivity, specificity, positive predictive value (PPV), and negative predictive value (NPV) were reported at a transparent operational predicted-CE threshold of ≥ 50%. All analyses were performed in R version 4.3.0. Model development used the caret and XGBoost packages, and SHapley Additive exPlanations (SHAP) values were used to interpret the final model [26]. Additional methodological details are provided in Supplementary Materials.

## Results

### Patient Characteristics and Baseline Demographics

A total of 206 patients who underwent leukapheresis were included. The cohort was characterized by a broad range of baseline hematologic variability, reflecting a real-world CAR-T candidate population (Table 1). The mean CE was 31.9% ± 21.1%. Consistent with the biological dependence of leukapheresis on the circulating T-cell burden, we observed a robust positive correlation between CE and precollection lymphocyte counts (*r* = 0.34, *P* < 0.001).

**Table 1:** Baseline characteristics and correlation with collection efficiency (CE)

Variable	Total Cohort (n = 206)	Correlation with CE (*r*)	*P* value/Adjusted *P* value
Demographics			
Age (years), mean (SD)	48.3 (16.0)	−0.28	<0.001
Body surface area (m^2^), mean (SD)	1.7 (0.2)	0.22	0.002
Male gender, n (%)	124 (60.2%)	–	0.180^†^
Hematologic parameters			
Precollection lymphocytes (×10⁹/L)	1.6 (2.5)	0.34	<0.001
Lymphocyte/WBC ratio	0.27 (0.18)	0.41	<0.001
Precollection monocytes (×10⁹/L)	0.61 (0.90)	0.19	0.007
Precollection WBC (×10⁹/L)	6.0 (4.7)	0.01	0.890
Observed CE classification^‡^			
Low efficiency (<30%), n (%)	89 (43.2%)	Reference	–
Moderate efficiency (30%–60%), n (%)	84 (40.8%)	–	–
High efficiency (≥60%), n (%)	33 (16.0%)	–	–

^†^Adjusted for height and weight. ^‡^Observed CE classification is presented for descriptive clinical triage purposes.

Notably, compared with the absolute lymphocyte count alone, the lymphocyte/WBC ratio was more strongly correlated with CE (*r* = 0.41; *P* < 0.001), suggesting that the “purity” of the peripheral immune environment is a critical determinant of yield. Age was significantly negatively correlated (*r* =  −0.28; *P* < 0.001), indicating that age-related immunosenescence affects collection feasibility.

With respect to anthropometric factors, while initial unadjusted comparisons suggested sex-based differences in CE, multivariate adjustment revealed that these differences were driven by BSA and weight differences rather than biological sex (adjusted *P* = 0.180). This finding supports the use of BSA-indexed protocols over sex-based stratification.

### Model Performance and Internal Validation

**Fig. 1:**
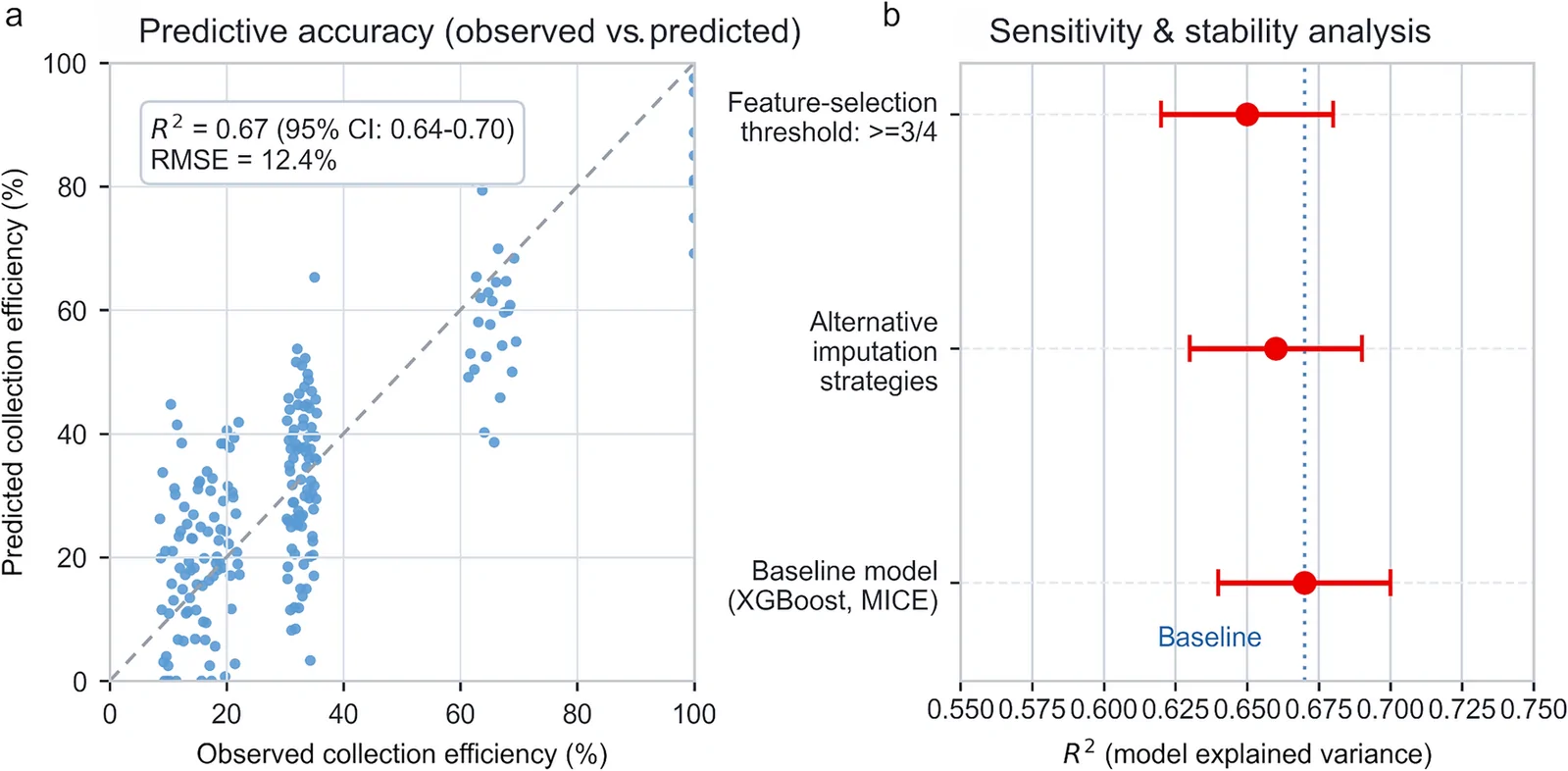
Model performance evaluation and robustness analysis. **a** Predicted versus observed mononuclear cell collection efficiency for the final XGBoost model. **b** Sensitivity analyses showing model performance under different missing-data strategies and feature-selection thresholds

We evaluated six supervised algorithms and compared the final model with a simple clinical baseline built from age, BSA, precollection lymphocyte count, and the lymphocyte/WBC ratio. XGBoost achieved an *R*^2^ of 0.67 (95% CI: 0.64–0.70), RMSE of 12.4%, MAE of 9.8%, and Pearson *r* of 0.82 (Table 2). The simple clinical baseline produced *R*^2^ = 0.245, RMSE = 18.3%, MAE = 13.6%, Pearson *r* = 0.498, and AUC = 0.846 for observed CE ≥ 60% (Table S5). Thus, XGBoost improved continuous CE estimation, whereas binary high-CE discrimination was similar between the simple baseline and XGBoost. Visual assessment of predicted versus actual collection efficiency confirmed close alignment along the identity line, with no systematic bias observed across the range of values (Figs. 1a, S2, and S3).

**Table 2:** Performance metrics of the final predictive model (XGBoost)

Metric	Value (95% CI)	Interpretation
*R*^2^ (explained variance)	0.67 (0.64–0.70)	Model explains about 67% of CE variability
RMSE (root mean square error)	12.4% (11.8–13.1)	Average prediction error magnitude
MAE (mean absolute error)	9.8% (9.3–10.4)	Average absolute deviation
Simple clinical baseline	*R*^2^ = 0.245; RMSE = 18.3%; MAE = 13.6%; *r* = 0.498; AUC = 0.846	Age, BSA, lymphocyte count, and lymphocyte/WBC ratio; tenfold OLS benchmark. AUC was similar to XGBoost, while continuous-error metrics were worse
Pearson *r*	0.82 (0.80–0.84)	Strong linear correlation with actuals
AUC (for CE ≥ 60%)	0.84 (0.78–0.90)	Secondary high-CE discrimination; 33/206 positive cases, so the CI is relatively wide
Sensitivity at predicted CE ≥ 50%	84.8% (28/33; approx. 95% CI, 69.1%–93.3%)	Estimated from the limited observed high-CE group
Specificity at predicted CE ≥ 50%	97.1% (168/173; approx. 95% CI, 93.4%–98.8%)	Estimated from observed non-high-CE cases
Positive predictive value	84.8% (28/33; approx. 95% CI, 69.1%–93.3%)	Probability of observed CE ≥ 60% among predicted positives
Negative predictive value	97.1% (168/173; approx. 95% CI, 93.4%–98.8%)	Probability of observed CE < 60% among predicted negatives

High CE was defined as an observed CE ≥ 60% (33/206). Threshold metrics at a predicted CE ≥ 50% were derived from 28/33 true positives and 168/173 true negatives; because the positive class was limited, these estimates should be interpreted with caution

Sensitivity analyses revealed minimal performance changes across missing-data strategies (MICE, listwise deletion, mean imputation, and random-forest imputation) and feature-selection thresholds (Δ*R*^2^ < 0.03; Fig. 1b, Table S3). In the ACD-adjusted CE sensitivity recalculation using the standard 12:1 whole blood:ACD assumption, the mean CE increased from 31.9% to 34.2%, and the median CE increased from 31.2% to 33.8%, but the low/moderate/high CE distribution remained at 89/84/33; no patient changed the CE stratum, and the number of uncapped CE values > 100% remained at eight (Table S6).

In the secondary clinical triage analysis, high CE was defined as a CE ≥ 60% and occurred in 33 of 206 patients (16.0%; Fig. S1). The continuous predicted CE score discriminated observed high CE, with an AUC of 0.84 (95% CI: 0.78–0.90). At the operational predicted-CE threshold of ≥ 50%, 28 of 33 high-CE cases were identified, and 168 of 173 nonhigh-CE cases were correctly classified, yielding a sensitivity of 84.8% (approximately 95% binomial CI, 69.1%–93.3%), specificity of 97.1% (93.4%–98.8%), PPV of 84.8% (69.1%–93.3%), and NPV of 97.1% (93.4%–98.8%). Because the high-CE endpoint included only 33 positive cases, these threshold-based estimates should be interpreted as internally validated triage indicators rather than fixed operational targets.

### Key Predictors and Clinical Interpretability

**Fig. 2:**
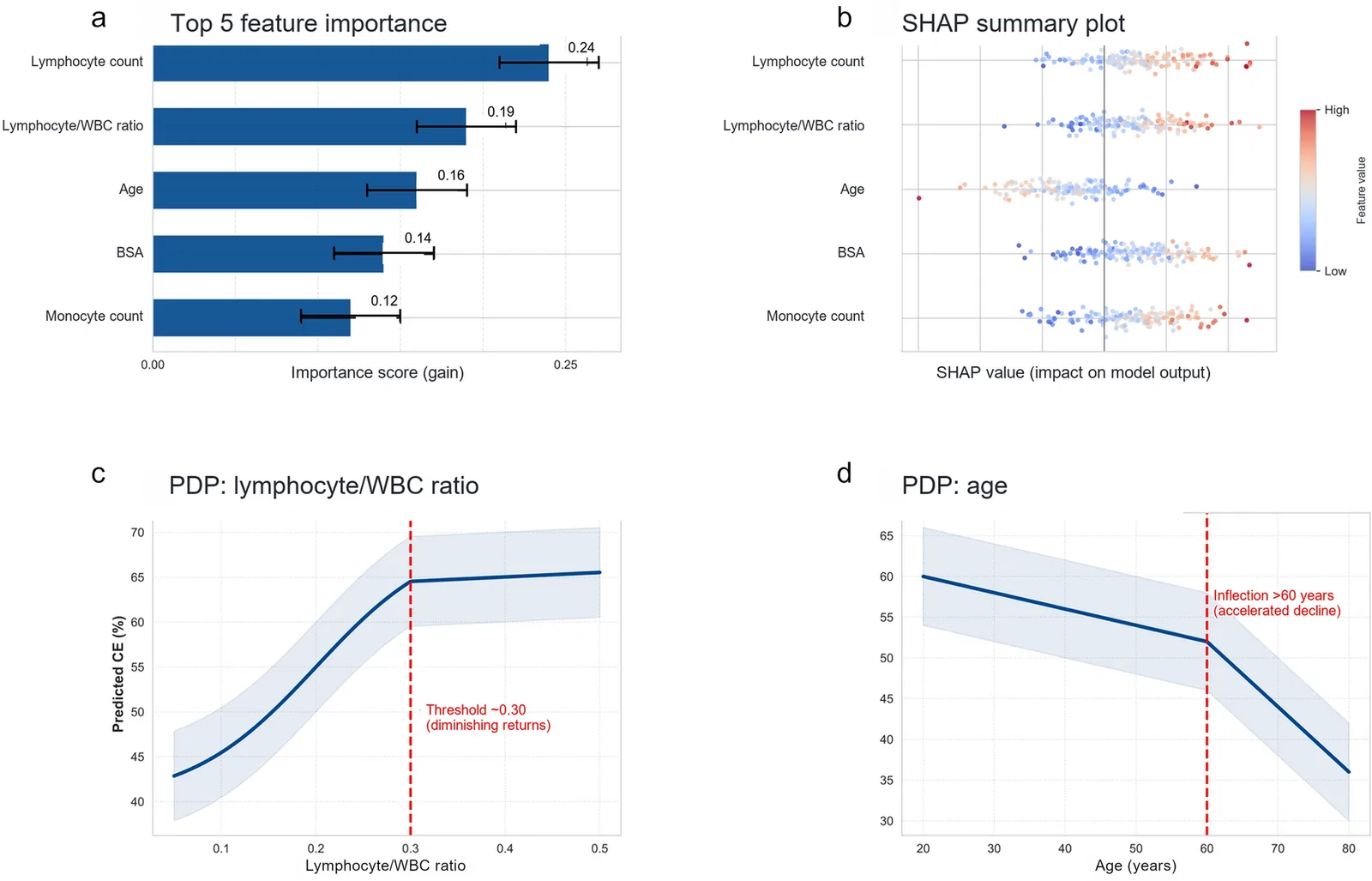
Feature importance and clinical interpretability. **a** Relative importance of the dominant predictors in the final model. **b** SHAP summary plot for the five dominant predictors. **c** Model-interpretability curve for lymphocyte/WBC ratio, showing a plateau after approximately 0.30. **d** Model-interpretability curve for age, showing a steeper predicted CE decline after approximately 60 years. Error bars and shaded intervals represent variability across the internal validation workflow

Feature importance analysis revealed that five predictors accounted for most of the model signal: lymphocyte count, lymphocyte/WBC ratio, age, BSA, and monocyte count (Fig. 2a).

Model interpretability analyses revealed clinically interpretable patterns (Fig. 2b–d):

1. The model interpretability plots indicated a nonlinear association between the lymphocyte/WBC ratio and CE, with predicted gains rising steeply until approximately 0.30 and then plateauing (Fig. 2c). This pattern suggests a practical reference point for precollection review, but it should not be treated as a validated clinical cutoff.
2. The predicted CE declined nonlinearly with age, with a more pronounced reduction after approximately 60 years (Fig. 2d). This age-related reference point is consistent with immune senescence, but it requires external validation before being used for protocol decisions.
3. The interaction pattern suggested that the BSA and lymphocyte count jointly contributed to the predicted yield; patients with higher BSA and adequate lymphocyte counts tended to have higher predicted CE.

### Sensitivity Analyses

Sensitivity analyses revealed stable performance across different imputation strategies and feature-selection thresholds, supporting the internal stability of the final model while not replacing external validation. The MICE, listwise deletion, mean imputation, and random-forest imputation analyses all preserved the same model ranking, with *R*^2^ values ranging from 0.65 to 0.69.

## Discussion

### Principal Findings

In this retrospective cohort of 206 patients, an interpretable XGBoost-based model estimated the MNC collection efficiency from routine precollection variables with good internal performance (*R*^2^ = 0.67; RMSE = 12.4%). The model remained stable across nested cross-validation, bootstrap resampling, and sensitivity analyses, but these findings are still internally validated. The strongest predictors were the precollection lymphocyte count, lymphocyte/WBC ratio, age, and BSA.

### Biological Plausibility and Interpretability

A common concern in clinical machine learning is that high-performing models may remain difficult for end users to interpret [27]. In the present study, however, the dominant predictors were biologically plausible and aligned with the established principles of leukapheresis. The precollection lymphocyte count emerged as the strongest predictor, which is consistent with prior evidence linking lymphocyte availability to collection yield [5].

Beyond absolute counts, the model highlighted the lymphocyte/WBC ratio as an informative index of collection conditions. In this cohort, the interpretability plots suggested diminishing returns above a lymphocyte/WBC ratio of approximately 0.30, indicating that relative lymphocyte availability, not only absolute counts, was associated with CE. The age curve showed a steeper decline after approximately 60 years, which is compatible with immune senescence [11]. These patterns should be interpreted as cohort-specific reference points for clinical review, not as externally validated thresholds. Prior CAR-T processing studies have also shown that the leukapheresis composition can influence T-cell activation and transduction efficiency [28], and T-cell fitness in autologous CAR-T therapy depends on a broader lymphocyte composition [29]. Basic apheresis principles support this interpretation because separator performance depends on the circulating cellular mixture [30]. Chronic inflammation and aging may further contribute to reduced immune cell reserves in vulnerable patients [31].

### Comparison with Previous Studies

Previous T-cell collection studies have relied primarily on predictive algorithms or mathematical modeling [9,10]. In the present cohort, the XGBoost model captured nonlinear patterns in continuous CE, although the simple clinical baseline showed a similar AUC for the secondary high-CE endpoint. The present analysis adds an internally validated estimate of continuous CE and an explicit risk-stratified summary for collection planning.

Those prior T-cell collection models typically assume fixed relationships between variables. The present model captured nonlinear patterns in age, lymphocyte/WBC ratio, BSA, and lymphocyte count (Fig. 2b–d). Compared with machine learning models developed for hematopoietic stem cell mobilization in healthy donors [12, 32], the present model was calibrated to a CAR-T candidate population, which typically comprises patients who are heavily pretreated and lymphopenic [4, 33].

### Clinical Implications

The clinical value of this framework lies in its potential to inform precollection planning rather than replace clinical judgment. A lymphocyte/WBC ratio of approximately 0.30 and an age of approximately 60 years may help clinicians identify cases that warrant closer review of timing, recent therapy exposure, and expected collection yield. To make the proposed workflow explicit, we grouped patients by predicted CE into three operational strata: predicted CE < 30%, 30%–60%, and ≥ 60%.

Patients in the predicted CE ≥ 60% stratum accounted for 20 of 206 cases (9.7%) and had a high observed CE profile (mean 77.8%, median 68.2%). For this group, standard leukapheresis protocols may be adequate, and the processing time or volume may be optimized.

Patients in the predicted CE 30%–60% stratum accounted for 80 cases (38.8%) and had intermediate observed CE (mean 34.5%, median 33.0%). These cases may be candidates for targeted optimization before collection.

For patients with a transiently suppressed lymphocyte/WBC ratio, recent therapies or growth factor exposure should prompt reassessment of collection timing and product composition rather than automatic extension of procedure time [7].

In this cohort, the interaction between BSA and yield suggested that BSA-adjusted processed blood volume could be explored, although direct CAR-T-specific evidence is limited, and the supporting evidence comes mainly from stem cell collection settings [34].

Patients in the predicted CE < 30% stratum accounted for 106 cases (51.5%) and had low observed CE (mean 21.2%, median 18.6%). Early identification of this group may facilitate contingency planning, including collection deferral, protocol adjustment, or, by analogy with hematopoietic progenitor cell apheresis [HPC(A)] collection practices, consecutive-day collection when clinically appropriate [35].

**Fig. 3:**
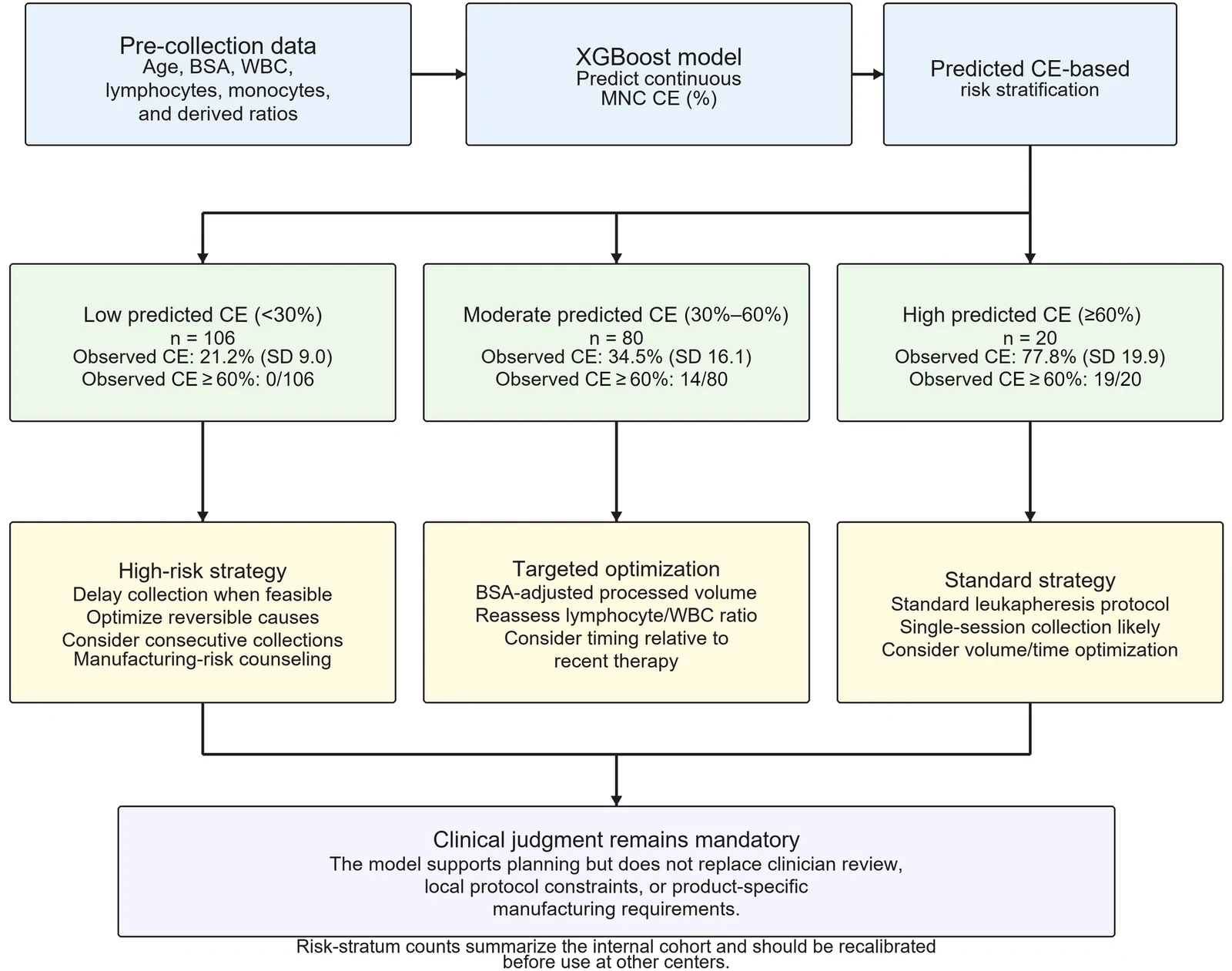
Proposed data-driven decision-support workflow for leukapheresis planning based on predicted collection efficiency. The schematic summarizes precollection triage according to predicted CE (< 30%, 30%–60%, and ≥ 60%), the observed patient distribution across strata, and corresponding planning strategies

More broadly, this workflow provides a quantitative framework for standardizing decisions that are often made empirically in leukapheresis practice (Fig. 3).

This three-tier scheme is therefore an operational summary of the model output, not a mandatory treatment algorithm: patients predicted to have a CE of 30 %–60% may be candidates for targeted optimization strategies, whereas those predicted to have a CE < 30% can be flagged early for additional planning and counseling.

Thus, the model is best viewed as a decision-support tool that complements, rather than supplants, clinician expertise.

### Limitations

We acknowledge several limitations. First, this was a retrospective single-center study, and external validity across institutions, patient populations, and apheresis platforms remains to be established [25]. Nested cross-validation, bootstrap resampling, and sensitivity analyses support internal validity but cannot replace prospective external validation; the modest sample size and feature-selection process may still produce optimistic performance estimates. Before routine clinical deployment, the proposed decision-support workflow should be evaluated prospectively with attention to workflow integration and human factors [36]. Second, the secondary high-CE analysis included only 33 positive cases. The AUC confidence interval was therefore relatively wide, and the sensitivity, specificity, PPV, and NPV at the predicted-CE ≥ 50% threshold should be interpreted with caution. Third, CE was used as the primary endpoint. Although the CE is operationally important, it does not directly capture the T-cell phenotype, fitness, transduction efficiency, manufacturing success, or clinical response [37]. Fourth, several potentially important confounders were unavailable or incompletely recorded, including disease subtype, prior lines of therapy, time since the last chemotherapy, recent granulocyte colony-stimulating factor exposure, steroid exposure, and detailed product-manufacturing parameters. Finally, the proposed workflow should be viewed as a decision-support framework requiring prospective calibration before routine clinical deployment.

## Conclusions

In conclusion, an interpretable machine learning model based on routine precollection variables was used to estimate MNC collection efficiency with good internal validity. The clarified CE definition, threshold-based high-CE triage metrics, and risk-stratified workflow may support individualized leukapheresis planning, but the observed reference points and threshold metrics require prospective multicenter validation before routine implementation.

## Supplementary Information

Below is the link to the electronic supplementary material.

Supplementary file1 (PDF 460 KB)

## Data Availability

The datasets generated during and/or analysed during the current study are available from the corresponding author on reasonable request.
