# Supplementary material for: Machine Learning-Based Prediction of Mononuclear Cell Collection Efficiency for CAR-T Manufacturing
Source: Curr Med Sci. 2026 Aug 5;46(4):1014–22. doi: 10.1007/s11596-026-00237-1 (PMC13503503; doi:10.1007/s11596-026-00237-1)
Supplement: Supplementary file 1 [file 11596_2026_237_MOESM1_ESM.pdf]

Supplementary Materials

Table S1. MICE imputation convergence diagnostics and pre/post-imputation distributions

Variable	Missing rate (%)	Imputation method	Convergence (trace plot)	Pre-imputation mean (SD)	Post-imputation mean (SD)	KS test *P*-value	Distribution shift (yes/no)
Pre-collection lymphocytes	2.4	Predictive mean matching	Stable	1.54 (2.51)	1.56 (2.50)	0.97	No
Lymphocyte/WBC ratio	2.4	Predictive mean matching	Stable	0.27 (0.18)	0.27 (0.18)	0.99	No
Age	0	-	-	48.3 (16.0)	48.3 (16.0)	-	No
Body surface area	1.0	Predictive mean matching	Stable	1.71 (0.20)	1.71 (0.20)	0.99	No
Pre-collection monocytes	3.4	Predictive mean matching	Stable	0.61 (0.91)	0.62 (0.91)	0.96	No
BMI	0.5	Predictive mean matching	Stable	23.4 (4.1)	23.4 (4.1)	0.98	No
Pre-collection HGB	2.9	Predictive mean matching	Stable	101.7 (31.2)	101.8 (31.2)	0.97	No
Disease category	0	-	-	-	-	-	-
Collection machine type	0.5	Multinomial logistic	Stable	-	-	-	No
Pre-collection PLT	1.9	Predictive mean matching	Stable	176.6 (98.2)	176.6 (98.2)	0.99	No

KS test evaluates the distributional difference before and after imputation. $P > 0.05$ indicates no significant distribution shift. The lymphocyte/WBC ratio is reported using the same analytic definition as the main manuscript (0.27 [0.18]).

Table S2. Complete hyperparameter search spaces for all algorithms

Model	Hyperparameter	Search space/values	Optimization method
Linear regression	-	-	-
Ridge regression	λ (L2 penalty)	[1e-4, 1e-3, 1e-2, 0.1, 1, 10]	Grid search
Lasso regression	λ (L1 penalty)	[1e-4, 1e-3, 1e-2, 0.1, 1, 10]	Grid search
Elastic net	α (L1/L2 ratio)	[0.1, 0.3, 0.5, 0.7, 0.9]	Grid search
	λ (regularization)	[1e-4, 1e-3, 1e-2, 0.1, 1, 10]	Grid search
Random forest	n_estimators (trees)	500	Fixed
	mtry (features per split)	[2, 4, 6, 8, 10]	Grid search
	nodesize (min leaf size)	[1, 3, 5]	Grid search
XGBoost	nrounds (boosting rounds)	[100, 200, 300, 500]	Grid search
	max_depth	[3, 6, 9]	Grid search
	eta (learning rate)	[0.01, 0.1, 0.3]	Grid search
	subsample	0.8	Fixed
	colsample_bytree	0.8	Fixed
Feature selection	Pearson screening	$P < 0.05$	Filter before ensemble vote
Feature selection	Boruta	500 trees; maxRuns = 500	Random-forest all-relevant selection
Feature selection	RFE	Repeated CV; RMSE scoring	Wrapper selection
Feature selection	Variance filter	Near-zero variance removed	Stability filter

All models were tuned using inner-loop cross-validation within nested 10-fold CV. The outer 10-fold loop was repeated three times for stability summaries, whereas the primary performance estimate was based on the nested CV framework described in the main manuscript.

Table S3. Sensitivity analysis results for different imputation methods

Imputation method	R^2 (mean, 95% CI)	RMSE (%) (mean, 95% CI)	MAE (%) (mean, 95% CI)	Pearson r (mean, 95% CI)	Model ranking change
MICE (default, PMM)	0.67 (0.64–0.70)	12.4 (11.8–13.1)	9.8 (9.3–10.4)	0.82 (0.80–0.84)	Baseline
Listwise deletion	0.65 (0.62–0.68)	12.7 (12.1–13.5)	10.0 (9.5–10.7)	0.80 (0.77–0.83)	No change
Mean imputation	0.66 (0.63–0.68)	12.6 (12.0–13.3)	9.9 (9.4–10.4)	0.81 (0.78–0.83)	No change
Random forest imputation	0.69 (0.66–0.71)	12.1 (11.6–12.8)	9.7 (9.2–10.3)	0.83 (0.81–0.85)	No change

Model performance was robust to different imputation strategies. Changes in R^2 were all <0.03 and model ranking remained stable.

Table S4. Complete list of candidate variables and feature selection results

Category	Variable name	Variable type	Selection frequency (max 4)*	Included in final model?
Demographics & anthropometry	Age	Continuous	4/4	Yes
	Body surface area (BSA)	Calculated	4/4	Yes
	Body mass index (BMI)	Calculated	3/4	Yes
	Sex (male/female)	Categorical	1/4	No†
	Weight	Continuous	2/4	No‡
	Height	Continuous	1/4	No‡
Hematology (pre-collection)	Lymphocyte count	Continuous	4/4	Yes
	Monocyte count	Continuous	4/4	Yes
	White blood cell (WBC) count	Continuous	3/4	Yes
	Platelet count (PLT)	Continuous	3/4	Yes
	Hemoglobin (HGB)	Continuous	3/4	Yes
	Hematocrit (HCT)	Continuous	2/4	No
	Neutrophil count	Continuous	2/4	No
Engineered ratios	Lymphocyte/WBC ratio	Calculated	4/4	Yes
	Platelet/WBC ratio	Calculated	3/4	Yes
	Hemoglobin/hematocrit ratio	Calculated	3/4	Yes
	Monocyte/WBC ratio	Calculated	2/4	No
Clinical & technical	Disease category (diagnosis)	Categorical	3/4	Yes
	Collection machine type	Categorical	3/4	Yes
	Prior lines of therapy	Incomplete; exploratory	2/4	No; exploratory only
	Time since last chemo	Incomplete; exploratory	2/4	No; exploratory only
Non-linear & interaction terms	Age^2 (polynomial term)	Engineered	4/4	Yes
	BSA × lymphocyte count	Interaction	3/4	Yes
	Age × lymphocyte count	Interaction	3/4	Yes
	BMI × BSA	Interaction	2/4	No
	Diagnosis × age	Interaction	1/4	No
	Diagnosis × lymphocyte	Interaction	1/4	No
	Machine × BMI	Interaction	1/4	No
Total features				13 base + 3 non-linear/interactions

Selection Frequency represents the number of feature selection methods (out of four: Pearson correlation, Boruta, RFE, and variance thresholding) that identified the variable as significant. Variables marked as incompletely recorded were assessed only as exploratory candidates and were not retained in the final model.
Feature-selection settings: Pearson screening used two-sided $P < 0.05$; Boruta used a 500-tree random forest with maxRuns = 500; RFE used repeated cross-validation with RMSE as the scoring criterion; and the variance filter removed near-zero-variance predictors.
Inclusion Criterion: Features selected by ≥3 methods were retained for final XGBoost model training. This table summarizes internal feature-selection stability; it should not be interpreted as external validation.

- † Sex: Excluded from the final model as multivariate analysis showed collection efficiency was driven by BSA and blood volume rather than biological sex (see Main Text, Results 3.1).
- ‡ Weight/Height: While measured, these were captured more effectively by the composite BSA variable and thus excluded as individual features to prevent collinearity.

Table S5. Simple clinical baseline benchmark against XGBoost

Model	Predictors / score	R^2	RMSE (%)	MAE (%)	Pearson r	AUC for CE ≥ 60%
Lymphocyte/WBC ratio alone	Single continuous predictor	0.130	19.6	14.3	0.365	0.739
Pre-collection lymphocyte count alone	Single continuous predictor	0.083	20.2	14.5	0.293	0.621
Age + lymphocyte/WBC ratio	Simple two-variable clinical model	0.209	18.7	13.8	0.459	0.792
Age + BSA + lymphocyte count + lymphocyte/WBC ratio	Prespecified simple clinical baseline; 10-fold OLS	0.245	18.3	13.6	0.498	0.846
XGBoost	Final model continuous predicted CE score	0.67	12.4	9.8	0.82	0.84

Baseline models were fit using the current 206-patient analytic dataset. The AUC column uses the continuous predicted CE score to classify observed CE ≥60%. The simple clinical baseline and XGBoost showed similar high-CE AUC values; the main advantage of XGBoost was better continuous CE estimation, reflected by higher R^2 and lower RMSE/MAE.

Table S6. ACD-adjusted collection-efficiency sensitivity recalculation

Measure	Primary CE	ACD-adjusted sensitivity	Interpretation
Assumption	Recorded clinical CE	Whole blood:ACD = 12:1	Patient-level ACD volume was not consistently recorded; sensitivity analysis only
Mean CE (%)	31.9	34.2	Small upward shift after denominator adjustment
Median CE (%)	31.2	33.8	Small upward shift after denominator adjustment
Low/moderate/high CE strata	89 / 84 / 33	89 / 84 / 33	No distributional change
Patients changing CE stratum	Reference	0	Risk-stratum conclusions unchanged
Uncapped CE >100%	8	8	Quality-control flag unchanged
Correlation with primary CE	Reference	0.998	Near-identical ranking

Note: ACD-adjusted CE was calculated as a sensitivity check by multiplying uncapped CE by 13/12 and then applying the same 100% cap used in the primary analysis. This sensitivity recalculation follows the CE2 denominator correction concept but was not used to replace the recorded clinical CE primary endpoint because patient-level ACD volumes were not consistently available.

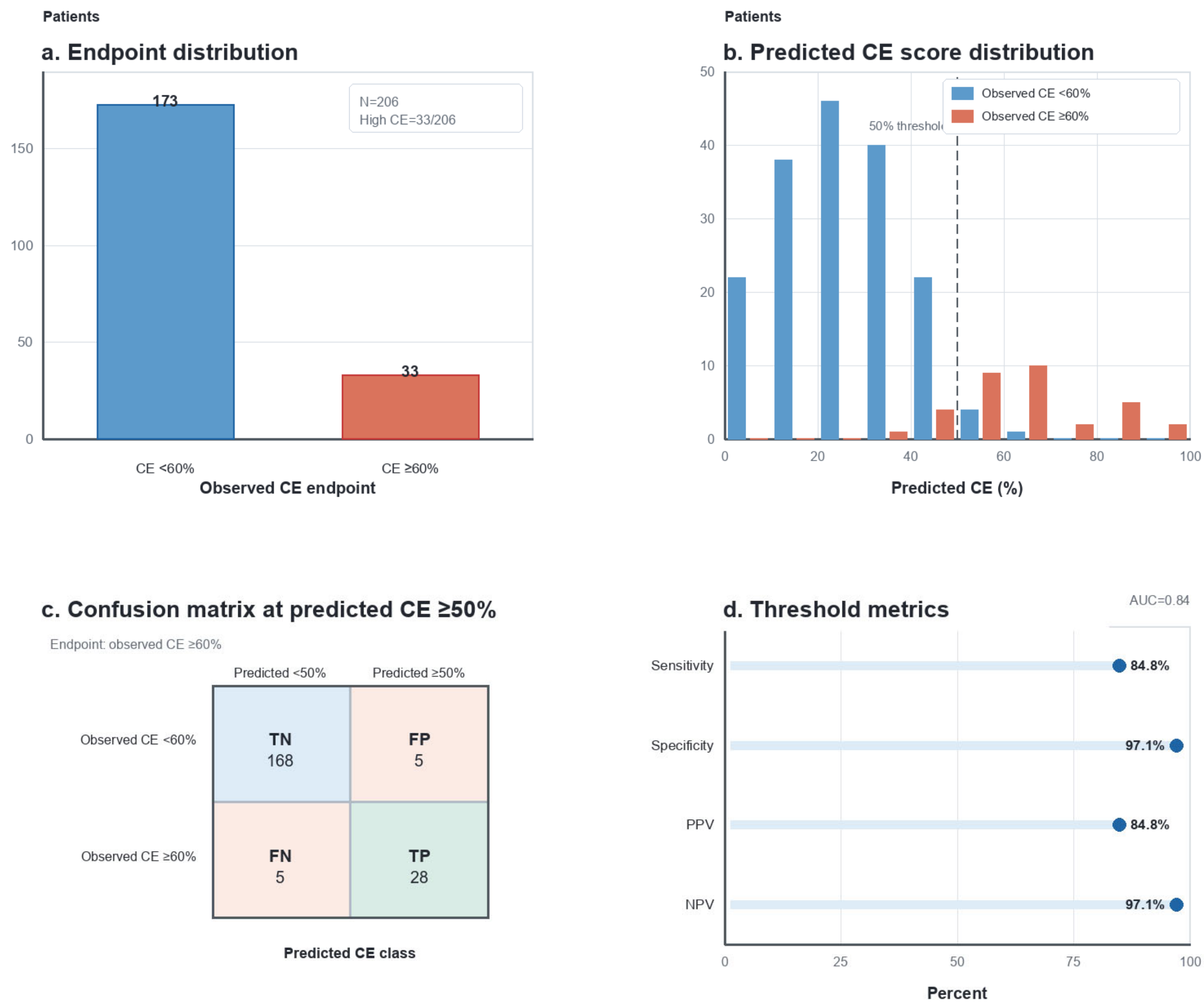

Fig. S1 High-efficiency triage performance using observed CE ≥ 60% as the binary endpoint. (**a**) Observed high CE was defined as CE ≥ 60% and occurred in 33 of 206 patients (16.0%), consistent with the main manuscript Table 1. (**b**) Predicted CE score distribution by observed endpoint class, with the operational predicted-CE threshold shown at ≥50%. (**c**) At the operational predicted-CE threshold of ≥50%, the model identified 28 of 33 high-CE cases and correctly classified 168 of 173 non-high-CE cases. (**d**) Threshold metric dot plot showing sensitivity 84.8%, specificity 97.1%, PPV 84.8%, and NPV 97.1%. The continuous predicted CE score discriminated observed CE ≥60% with an AUC of 0.84 (95% CI: 0.78-0.90).

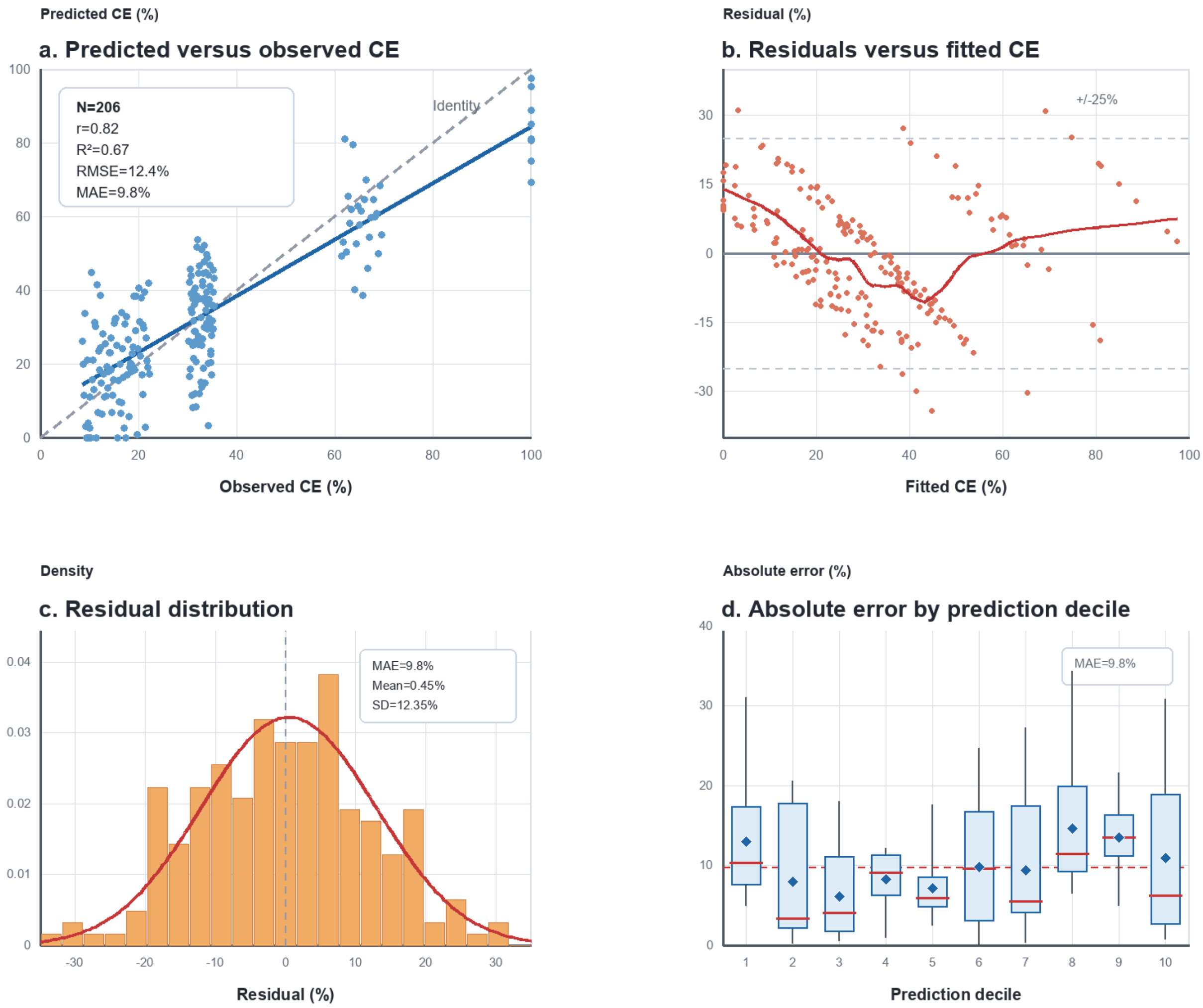

Fig. S2 Prediction-error diagnostics for continuous collection-efficiency estimation. (**a**) Predicted versus observed CE for the final XGBoost model, with identity and fitted trend lines and the reported internal validation metrics. (**b**) Residuals plotted against fitted CE, with the zero-residual reference, +/-25% residual bands, and LOESS smoothing to assess residual structure. In this diagnostic panel, residuals are displayed as observed CE minus predicted CE. (**c**) Residual distribution with normal-reference overlay, summarized by MAE=9.8%, mean residual=0.45%, and SD=12.35%; residuals are displayed as observed CE minus predicted CE. (**d**) Absolute prediction error by prediction decile; the dashed line marks the overall MAE=9.8%, and diamonds mark decile means.

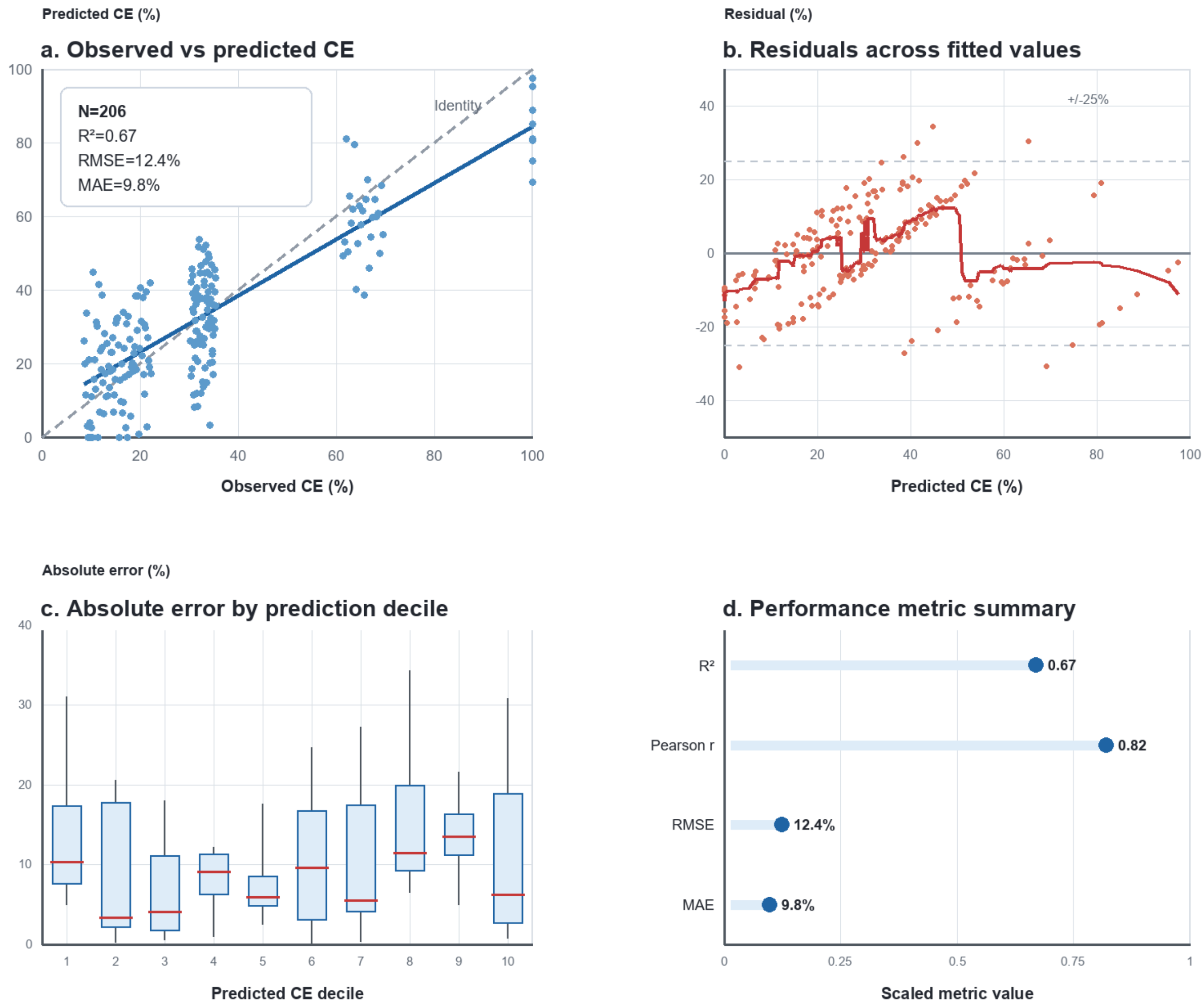

Fig. S3 Continuous collection-efficiency model validation. (**a**) Observed versus predicted mononuclear cell collection efficiency for the final XGBoost model in the 206-patient analytic cohort. (**b**) Residuals plotted against predicted CE, with the zero-residual reference and +/-25% residual bands. In this validation panel, residuals are displayed as predicted CE minus observed CE. (**c**) Absolute prediction error summarized across predicted-CE deciles to assess error stability over the prediction range. (**d**) Performance metric dot plot for continuous CE prediction, reporting R^2, RMSE, MAE, and Pearson correlation using the manuscript source-of-truth metrics. This figure evaluates continuous CE prediction only; high-CE triage metrics are reported separately in Fig. S1.
